# CT features associated with contralateral recurrence of spontaneous pneumothorax

**DOI:** 10.1093/qjmed/hcae129

**Published:** 2024-07-08

**Authors:** L A Burn, M T Wetscherek, P D Pharoah, S J Marciniak

**Affiliations:** Respiratory Medicine, Addenbrooke's Hospital, Cambridge University Hospitals NHS Foundation Trust, Cambridge, UK; Respiratory Medicine, Addenbrooke's Hospital, Cambridge University Hospitals NHS Foundation Trust, Cambridge, UK; Department of Computational Biomedicine, Cedars-Sinai Medical Center, Los Angeles, CA, USA; Respiratory Medicine, Addenbrooke's Hospital, Cambridge University Hospitals NHS Foundation Trust, Cambridge, UK; Cambridge Institute for Medical Research (CIMR), University of Cambridge, Cambridge, UK; Respiratory Medicine, Royal Papworth Hospital, Cambridge, UK

## Abstract

**Introduction:**

Spontaneous pneumothorax recurs in 30–54% of patients without surgery. Identifying individuals likely to suffer a recurrence, who might benefit from pre-emptive surgery, is challenging. Previous meta-analysis suggested a relationship between contralateral recurrence and specific CT findings.

**Methods:**

We analysed CT images and recurrence rates of 243 patients seen by our tertiary referral pneumothorax service.

**Results:**

We validated the meta-analysis observation that contralateral lung cysts are associated with a higher risk of contralateral recurrence in younger individuals. Furthermore, we observed that the size of contralateral cysts to be associated with increased contralateral recurrence in younger patients.

**Conclusion:**

The detection of contralateral lung cysts might therefore help identify younger patients more likely to benefit from pre-emptive surgery.

## Introduction

Spontaneous pneumothorax frequently presents to respiratory services.^1^ Depending on the context, initial management can be conservative observation, pleural aspiration or intercostal chest drainage.^2–4^ If a persistent air leak occurs, or if the pneumothorax recurs, surgical intervention is recommended.^4–6^ Predicting who will suffer a recurrence and might therefore benefit from pre-emptive surgery remains challenging.

The risk of recurrence following a first spontaneous pneumothorax in adults ranges from 30% to 54%, with ipsilateral recurrence being 2.3-fold more common than contralateral.^1^^,^^7–12^ Following any recurrence, current guidance recommends surgery.^4–6^^,^^13^ The ability to predict those most at risk of recurrence might improve treatment pathways, but effective risk predictors do not yet exist. Current clinical pathways do not advocate for routine thoracic CT scanning in pneumothorax, but our recent meta-analysis of 2475 individuals found that patients with abnormal scans were 2.5-times more likely to suffer a recurrence compared to those without.^7^ Strikingly, contralateral recurrence was strongly associated with contralateral CT change with an odds ratio of 8.1. We, therefore, set out to validate these findings in patients seen in our pneumothorax service over a 7-year period.

## Materials and methods

### Patient cohort

We analysed consecutive patients with a radiological diagnosis of pneumothorax attending our tertiary referral hospital between 12 November 2014 and 02 February 2022 (*n* = 343). Electronic notes were reviewed until data freeze on 13 February 2023. Individuals with pneumothorax in the context of trauma, infection, thoracic endometriosis, malignancy, interstitial lung disease or congenital abnormality were excluded (*n* = 42). The first recurrence was noted as being ipsilateral or contralateral relative to the first pneumothorax. Patients for whom the affected side could not be verified (*n* = 11) or when simultaneous bilateral pneumothoraces occurred (*n* = 3) were also excluded. Remaining patients with idiopathic pneumothoraces or pneumothoraces in the context of smoking or a variety of less common conditions including Birt–Hogg–Dubé syndrome, Marfan syndrome, vascular Ehlers–Danlos syndrome or alpha1-antitrypsin deficiency were considered (*n* = 287). Only thoracic CT scans performed after the initial pneumothorax were analysed. When CT scans were of insufficient quality owing to lobar collapse due to pneumothorax, then the next available scan was analysed. Patients with no CT scan (*n* = 43) or with inadequate thoracic coverage (*n* = 1) were excluded. The demographic and clinical data for the remaining patients are tabulated (*n* = 243; Table 1). Individuals excluded for having no CT were predominantly males (*n* = 40, 93%, *P* = 0.036), were younger (median age 21.4 vs. 30.2 years, *P* < 0.001) and were less likely to have a positive family history of pneumothorax (2.3% vs. 15%, *P* = 0.045; Supplementary Table S1). In the excluded group, more were smokers (37% vs. 29%, *P* = 0.038) but had fewer pack-years.

**Table 1.** Demographics table of cohort

	No recurrence	Recurrence	Chi^2^ test
	(*n* = 110)	(*n* = 133)	(*P* value)
Male sex—*N* (%)	86 (78%)	103 (77%)	1.00
Age—median (range)	32.5 (15.8-95.1)	27.7 (13.4-89.4)	**0.027** ^b^
Deceased^c^—*N* (%)	6 (5.5%)	0 (0%)	**0.021**
Smoking status—*N* (%)			
Ever	71 (65%)	71 (53%)	0.10
Current	30 (27%)	40 (30%)	0.13
Smoking type—*N* (%)			
Tobacco	69 (63%)	71 (53%)	0.18
Cannabis	43 (39%)	33 (25%)	**0.021**
Crack cocaine	1 (0.9%)	0 (0%)	0.9^a^
Hooka	0 (0%)	1 (0.8%)	0.9^a^
Smoking pack-years—mean (range)	10.07 (0–75)	8.28 (0–96)	0.17^b^
Missing data—*N* (%)	12 (11%)	6 (4.5%)	–
Initial treatment—*N* (%)			
Conservative	29 (26%)	41 (31%)	0.53
Aspirated	15 (14%)	20 (15%)	0.90
Thoracic drain	50 (45%)	56 (42%)	0.69
Surgery	16 (15%)	11 (8.2%)	0.18
Missing data	0 (0%)	5 (3.8%)	–
Family history^d^—*N* (%)	10 (9.1%)	26 (20%)	**0.035**
First pneumothorax—left/right	56 (51%)/54 (49%)	73 (55%)/60 (45%)	0.62
Recurrence—*N* (%)			
Left/right	–	67 (50%)/66 (50%)	–
Ipsilateral/contralateral	–	99 (74%)/34 (26%)	–
Pulmonary diagnosis—*N* (%)			
Birt–Hogg–Dubé syndrome	1 (0.9%)	4 (3.0%)	0.49
Marfan syndrome	1 (0.9%)	1 (0.8%)	1.00
A1AT deficiency	2 (1.8%)	0 (0%)	0.40
Vascular Ehlers–Danlos syndrome	1 (0.9%)	0 (0%)	0.92
Other^e^	4 (3.6%)	4 (3.0%)	–

a Yates’ continuity correction not applied.

b Mann–Whitney test.

c Deaths occurring during the study follow-up period.

d Affected 1st to 3rd degree relative.

e Other diagnoses include Behçet’s disease, congenital diaphragmatic hernia, idiopathic large pulmonary cyst, Loeys–Dietz syndrome, connective tissue disease.

A1AT = α_1_-antitrypsin deficiency.

*P* values <0.05 are highlighted bold.

### Ethics

The study was approved by the University of Cambridge Human Biology Research Ethics Committee HBREC.2020.28.

### Imaging analysis

Patients were grouped by the sequencing of their CT relative to the recurrent pneumothorax. Whenever possible, CT scans prior to the first recurrence were selected (*n* = 43, 32%); when patients underwent thoracic CT imaging only after their first recurrence, the first available scan was selected (*n* = 90, 68%); for patients with no recurrence, the first post-pneumothorax scan was selected (*n* = 110). All CT scans were then reviewed independently by two members of the research team (LAB and MTAW) including a consultant thoracic radiologist with 10 years of experience in reading thoracic CTs. Results for each lung were recorded separately for every patient.

### Imaging acquisition

Thoracic CT scans were performed across 22 different hospitals, with the majority conducted at a tertiary referral university hospital (*n* = 206, 85%). Scans were produced on one of 33 thin-section CT scanners by SIEMENS (two Somatom Definition Flash, four Somatom Definition AS+, three Somatom Definition AS, three Somatom Definition Edge, one Somatom Force, one Sensation 16, one Sensation 64), Canon Medical Systems (two Aquilion, four Aquilion Prime, one Aquillion One), GE MEDICAL SYSTEMS (two Revolution Evo, one Revolution CT, two Optima CT660, one Discovery MI DR, one Discovery CT750 HD, one Discovery 710) or Philips (one Brilliance 64, one Ingenuity Core 128, one iCT 256). The scanning protocol used volumetric acquisition from lung apices to the diaphragm with breath-hold at the end of deep inspiration. All examinations were reconstructed into continuous slices of thickness 1.0–2.0 mm by means of iterative reconstruction. All analysis was performed on the lung window series for all CT scans.

Emphysema was categorized according to the Fleischner Society classification system.^14^ The presence of paraseptal emphysema and centrilobular emphysema were noted, the latter graded from mild to moderate to severe. The number of cysts/blebs/bullae was recorded as ordinal values: 0 (none), 1 (*n* = 1–4) or 2 (*n* ≥ 4). The largest cyst in each lung was recorded in millimetres. Parenchymal and subpleural cysts were both considered in the analysis of cyst number and largest size. Blebs (<1 cm diameter) and bullae (>1 cm diameter) were defined as thin-walled spaces of air within the visceral pleura. Paraseptal emphysema was defined as well-demarcated juxta-pleural lucencies aligned in a row along the pleural margin.^14^^,^^15^ Centrilobular emphysema was defined as a centrilobular pattern of lucencies, and severity was graded depending on the extent of lung involvement. Cystic lung disease was defined as non-emphysematous cystic lesions, most often associated with a diagnosis. Throughout, the term ‘cyst’ incorporates blebs, bullae, subpleural cysts and cystic lung disease lesions.^15^

### Statistical analysis

Mann–Whitney *U* tests were used to analyse continuous variables with non-normal distribution. Chi-square test was used for comparison of categorical variables with Yates’ continuity correction applied. Cox regression analysis was used to investigate the risk factors for pneumothorax recurrence using hazard ratios. Follow-up started on the date of occurrence of first pneumothorax and finished on the date of a recurrent pneumothorax or on 13 February 2023 when all observations were censored. Factors with a *P* values <0.20 in univariable analysis were candidates for multivariable analysis. Backward elimination was used in multivariable analyses; only factors with a *P* values <0.05 were included in the final model. A *P* value <0.05 was considered statistically significant. Patients were analysed by recurrence status, smoking history and age. A stratifier of 50 years of age was chosen to reflect clinical guidelines that commonly use this age in definitions of secondary pneumothorax independent of the presence of thoracic CT changes.^4^ Data were analysed using R software^16^ implemented in R studio (version 2023.06.1 + 524)^17^ using the packages *Tidyverse*^18^ and *Meta*^19^ and all relevant code is available in the GitHub repository: https://github.com/LanceBn/CT-pneumothorax-retrospective-study.

## Results

### Patient cohort

**Figure 1.**
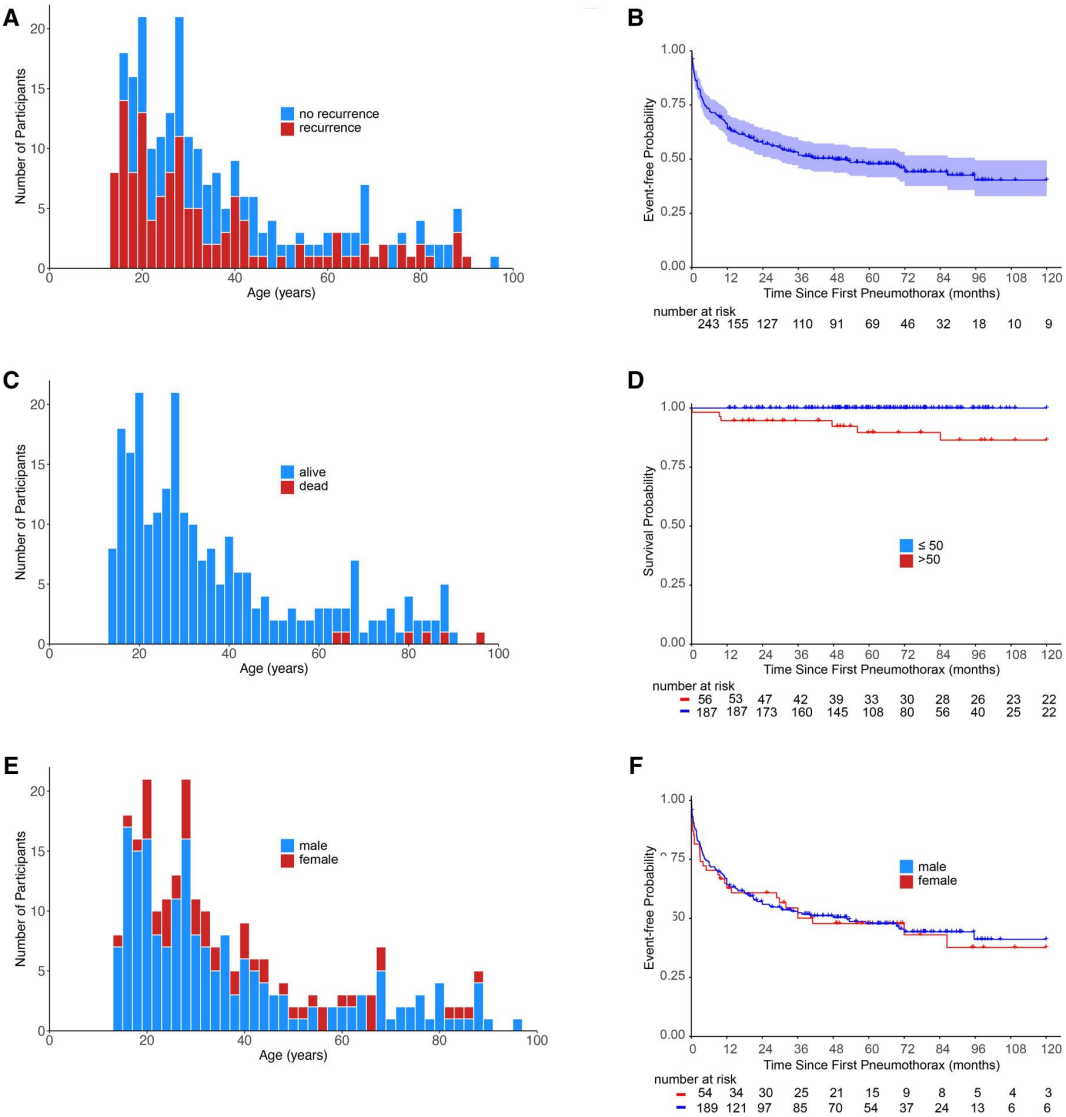
Patient cohort. (**A**) Stacked histogram plot of patients by age illustrating those who experience recurrence (red) or no recurrence (blue) at the time of analysis. (**B**) Kaplan–Meier survival curves illustrating proportion without recurrence over time in months. Shading illustrates 95% confidence interval. Crosses indicate censored points. Censored at 120 months. (**C**) Stacked histogram plot of patients by age illustrating those who had died (red) or were alive (blue) at the time of analysis. (**D**) Kaplan–Meier survival curves illustrating proportion alive over time in months, stratified by age: ≤50 years (blue), >50 years (red). Crosses indicate sanctioned points. Censored at 120 months. (**E**) Stacked histogram plot of patients by age illustrating females (red) and males (blue). (**F**) Kaplan–Meier survival curves illustrating proportion without recurrence over time in months, stratified sex. Crosses indicate censored points. Censored at 120 months.

A total of 243 patients met our criteria for inclusion of whom 133 had suffered at least one recurrence (55%, Table 1 and Fig. 1A and B). Recurrences were more common ipsilateral to the first pneumothorax (*n* = 99 [74%], Table 1). The groups were similar in sex, smoking status and initial management, but the non-recurrers were older than recurrers (median 32.5 vs. 27.7 years, *P* = 0.027; Table 1). Conservative management was common for the first pneumothorax (26% non-recurrers and 31% recurrers, *P* = 0.53). In each group at the first pneumothorax, similar proportions were treated by aspiration (14% non-recurrers vs. 15% recurrers) or intercostal chest drain (45% vs. 42%). Primary surgery was uncommon (15% in non-recurrers vs. 8.2% in recurrers, *P* = 0.18). The non-recurrence group included six individuals who had died by the time of data freeze, while none of the recurrent group had died (Table 1 and Fig. 1C and D). Smoking status was similar between the groups: 65% of non-recurrers vs. 53% of recurrers had ever smoked (*P* = 0.10); 27% of non-recurrers vs. 30% of the recurrers were current smokers (*P* = 0.13). Pack-years did not differ between the two groups (*P* = 0.17). However, non-recurrers included proportionately more cannabis smokers (39% vs. 25%, *P* = 0.021). Twice as many recurrers reported a family history of pneumothorax (9.1% non-recurrers vs. 20% recurrers, *P* = 0.035).

There was a total of 1670 person-years of follow-up with a median of 5.34 years. Univariable Cox regression analysis revealed family history of pneumothorax to be associated with overall recurrence risk (HR 1.77, 95% CI 1.14–2.73, *P* = 0.010, Supplementary Table S2). A history of cannabis smoking (HR 0.66, 95% CI 0.44–0.98, *P* = 0.040) and cigarette pack-years (HR 0.99, 95% CI 0.97–1.00, *P* = 0.040) were also associated with overall recurrence. Neither patient age (HR 0.99, 95% CI 0.98–1.00, *P* = 0.058) nor initial management with surgery (HR 0.49, 95% CI 0.23–1.05, *P* = 0.066) showed a significant association with overall recurrence. Multivariable Cox regression analysis of features predicting overall recurrence failed to generate a model in which more than one feature had a *P* values <0.05. This changed when the laterality of recurrence (ipsilateral vs. contralateral) was considered. For ipsilateral recurrence, a history of cannabis smoking and initial pneumothorax management with surgery were both associated with reduced recurrence risk on univariable and multivariable analyses (Supplementary Table S5). For contralateral recurrence, smoking pack-years, family history and patient age were all statistically significant; patient age continued to show significance in the multivariable analysis model (Supplementary Tables S6 and S7).

### CT changes

**Figure 2.**
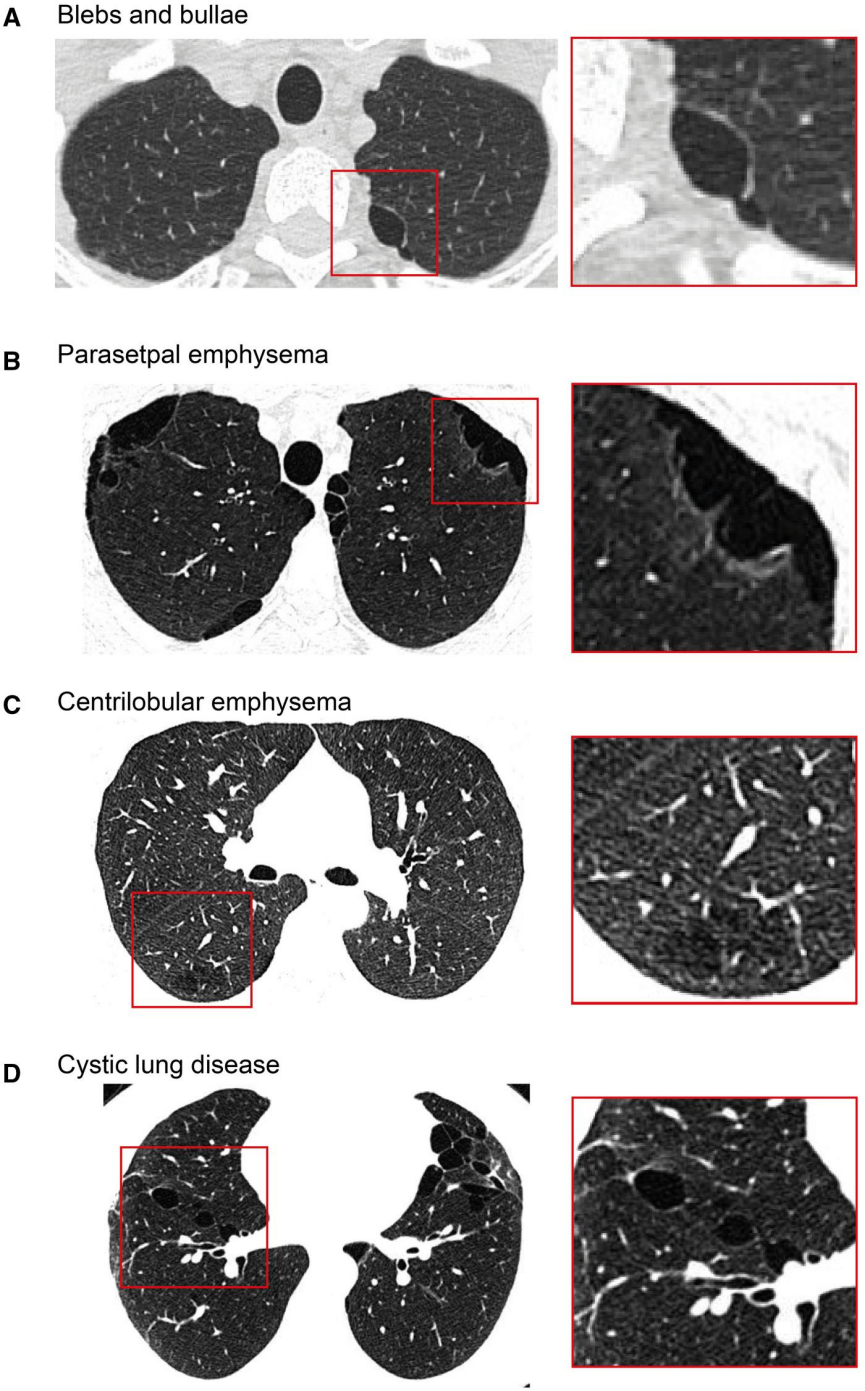
Example CT findings. (**A**) Isolated blebs and bulla in a non-smoker showing avascular low-attenuation thin-walled gas-containing spaces within the visceral pleura in the postero-medial aspect of the left lung apex. (**B**) Paraseptal emphysema in a tobacco and cannabis smoker showing multiple well-demarcated areas of subpleural emphysema along the parietal and mediastinal pleural margins. (**C**) Mild centrilobular emphysema in a tobacco and cannabis smoker showing a small region of centrilobular lucencies but predominated by large regions of normal lung. (**D**) Cystic lung disease is a never-smoker with Birt–Hogg–Dubé syndrome showing multiple simple and multiseptated cysts predominantly in the lower pulmonary zones.

Fleischner Society classification of CTs differed between never and ever smokers (Table 2). Examples of blebs/bullae, paraseptal and centrilobular emphysema, and cystic lung disease are presented (Fig. 2). Never-smokers were more likely to have no abnormality (ipsilateral 31% vs. 7.7%, *P* < 0.001; contralateral 40% vs. 11%, *P* < 0.001) or only isolated blebs/bullae (ipsilateral: 56% vs. 15%, *P* < 0.001; contralateral 49% vs. 9.2%, *P* < 0.001). Emphysematous change was more common in ever-smokers, both ipsilateral (77% vs. 5.9%, *P* < 0.001) and contralateral (80% vs. 5.9%, *P* < 0.001) to the first pneumothorax. No differences in these parameters were detected between the lungs of ex-smokers and current smokers (Table 3). Lung cysts (including blebs, bullae and cystic lung disease lesions^15^) were common in our cohort with 84% of cases having at least one. More cysts were detected in either lung of younger ever-smokers (ipsilateral, *P* = 0.002; contralateral, *P* = 0.005). In patients over 50 years, only cysts in the contralateral lung were associated with smoking (ipsilateral, *P* = 0.66; contralateral, *P* = 0.04). When present, lung cysts were commonly seen in isolation in the lungs of younger patients (80.3%). By contrast, isolated cystic change in those over 50 years was less common but rather as a component of emphysema (69.9%).

**Table 2.** CT changes in never and ever smokers

Parenchymal change	Never smoker	Ever smoker	Chi^2^ test
	(*n* = 101)	(*n* = 142)	(*P* value)
Ipsilateral—*N* (%)			
Isolated blebs/bullae	57 (56%)	21 (15%)	**<0.001**
Emphysema	6 (5.9%)	109 (77%)	**<0.001**
Cystic lung disease	7 (6.9%)	1 (0.7%)^a^	**0.021**
No abnormality	31 (31%)	11 (7.7%)	**<0.001**
Contralateral—*N* (%)			
Isolated blebs/bullae	49 (49%)	13 (9.2%)	**<0.001**
Emphysema	6 (5.9%)	113 (80%)	**<0.001**
Cystic lung disease	6 (5.9%)	1 (0.7%)^a^	**0.044**
No abnormality	40 (40%)	15 (11%)	**<0.001**

a This patient also has centrilobular and paraseptal emphysema.

Emphysema = paraseptal &/or centrilobular emphysema; cystic lung disease = patients with genetic disease that presented with lung cysts (i.e. mostly BHD).

Changes seen radiologically in the lungs ipsilateral and contralateral to the first pneumothorax. *P* values <0.05 are highlighted bold.

**Table 3.** CT changes in ex-smokers and current smokers

Parenchymal change	Ex-smoker	Current smoker	Chi^2^ test
	(*n* = 72)	(*n* = 70)	(*P* value)
Ipsilateral—*N* (%)			
Isolated blebs/bullae	11 (15%)	10 (14%)	1.00
Emphysema	54 (75%)	55 (79%)	0.76
Cystic lung disease	0 (0%)	1 (1.4%)^a^	0.99
No abnormality	7 (9.7%)	4 (5.7%)	0.56
Contralateral—*N* (%)			
Isolated blebs/bullae	5 (6.9%)	8 (11%)	0.53
Emphysema	58 (81%)	55 (79%)	0.93
Cystic lung disease	0 (0%)	1 (1.4%)^a^	0.99
No abnormality	9 (13%)	6 (8.6%)	0.63

a This patient also has centrilobular and paraseptal emphysema.

Emphysema = paraseptal &/or centrilobular emphysema; cystic lung disease = patients with genetic disease that presented with lung cysts (i.e. mostly BHD).

Changes seen radiologically in the lungs ipsilateral and contralateral to the first pneumothorax.

In our cohort, 76 individuals smoked or had smoked cannabis, 65 smoked or had smoked only tobacco, while 101 were never-smokers. Most cannabis smokers (*n* = 74, 97%) also smoked tobacco, though tobacco pack-years were lower in cannabis smokers compared to tobacco-only smokers (mean 8.89 vs. 25.6, *P* < 0.001, Supplementary Table S8). Cannabis smokers were younger than tobacco-only smokers (mean 29.4 vs. 63.1, *P* < 0.001). The absolute frequency of emphysema was similar in cannabis smokers and tobacco-only smokers (ipsilateral 76% vs. 83%, contralateral 76% vs. 83%) but the nature of the emphysema differed between the two groups. Paraseptal emphysema was more common in cannabis smokers compared with tobacco-only smokers (ipsilateral 75% vs. 55%, *P* = 0.014; contralateral 74% vs. 54%, *P* = 0.023, Supplementary Table S9). Conversely, centrilobular emphysema was less common in cannabis smokers compared with tobacco-only smokers (ipsilateral 33% vs. 72%, *P* < 0.001; contralateral 36% vs. 77%, *P* < 0.001).

### Association between CT changes and recurrence of pneumothorax

No association was observed between centrilobular nor paraseptal emphysema and recurrence (Supplementary Tables S2–S4). The presence of lung cysts did not associate with overall recurrence (hazard ratio [HR] 1.13, 95% CI 0.70–1.85, *P* = 0.61).

**Figure 3.**
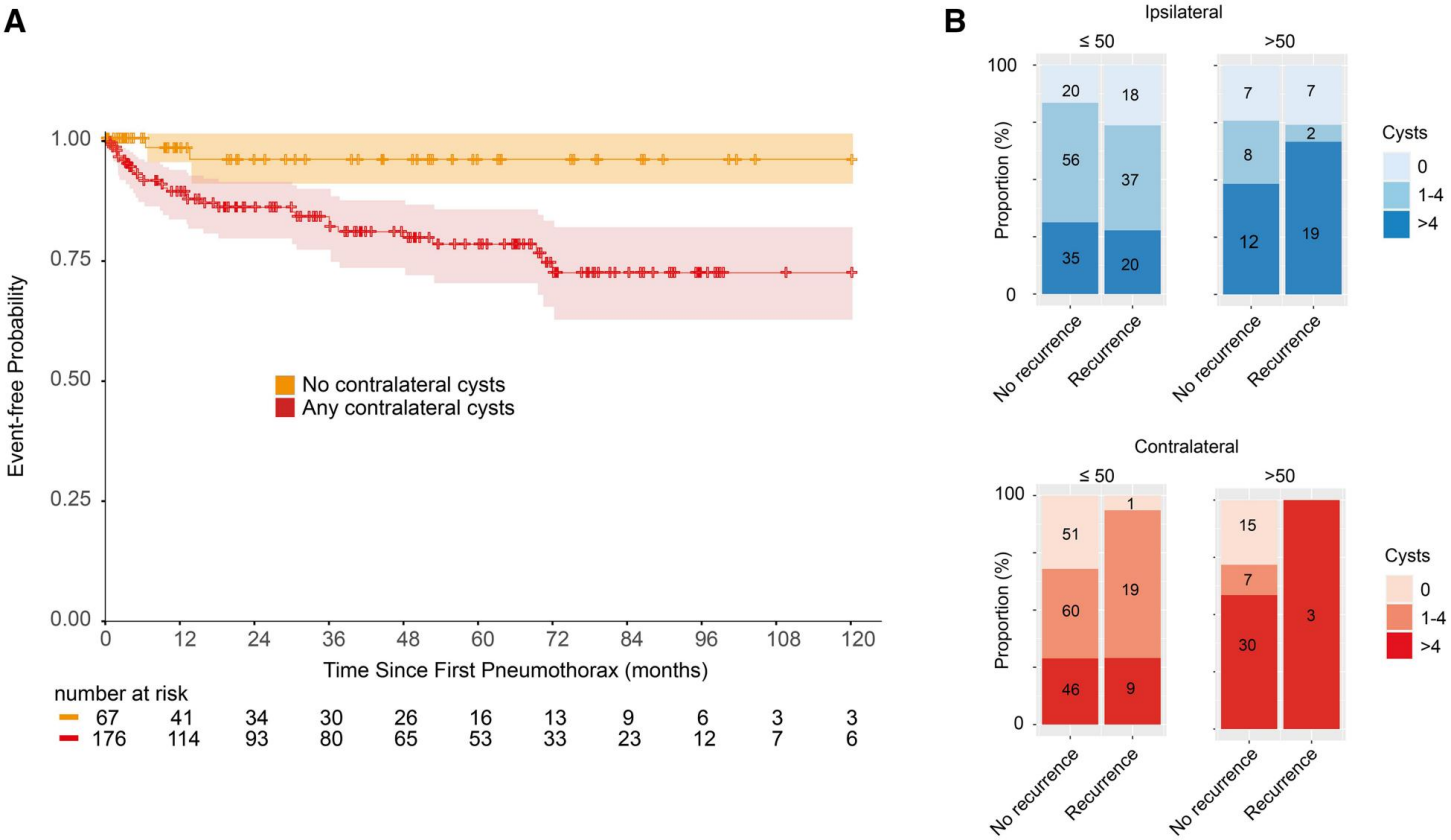
Lung cysts and their association with pneumothorax recurrence. (**A**) Kaplan–Meier survival curves illustrating event-free probability of contralateral recurrence over time in months for no contralateral cysts (orange) and any contralateral cysts (red). Crosses indicate censored points. Censored at 120 months. (**B**) Proportional stacked bar graphs showing pulmonary cyst number in lungs ipsilateral and contralateral to the first pneumothorax, stratified by laterality-specific recurrence and age.

When considered by their laterality, ipsilateral cysts failed to associate with ipsilateral recurrence, but contralateral cysts were associated with contralateral recurrences in both univariable and multivariable analyses (1–4 cysts, HR 7.07, 95% CI 1.65–30.3, *P* = 0.008; >4 cysts, HR 5.44, 95% CI 1.17–25.2, *P* = 0.03, Fig. 3, Supplementary Tables S6 and S7). When stratified by age, this association with contralateral recurrence was found to arise among younger individuals (*P* = 0.007), with no significant association detected in those over 50 years (*P* = 0.67, Fig. 3B). No association was seen with cyst size and ipsilateral recurrence (*P* = 0.58), but larger cyst size in the contralateral lung was associated with contralateral recurrence (recurrence median cyst size = 12 mm, range = 0–43mm; non-recurrers median = 5, range = 0–156; *P* = 0.002, Supplementary Table S6).

Receiver operator curve analysis generated a Youden’s threshold of 10.5 mm for largest cyst size (AUC 0.683, HR 2.75, 95% CI 1.33–5.66, *P* = 0.006). Of the 157 patients without contralateral pneumothorax, 42 (27%) had contralateral cysts ≥10.5 mm. Of the 30 individuals with contralateral recurrence, 17 (57%) had contralateral cysts ≥10.5 mm. This equates to a specificity of 0.73 and sensitivity was 0.57. Therefore, considering false and true positives, if contralateral cysts ≥10.5 mm were to be used to dictate contralateral surgery then 42/59 (71%) would have ‘unnecessary’ surgery (would not have suffered a recurrence), whereas 17/59 (29% would have suffered a recurrence).

## Discussion

We set out to validate the findings of our previous meta-analysis of the international literature, which had revealed an association between emphysematous features identified on thoracic CT and contralateral recurrence.^7^ In our single centre study, we confirm that lung cysts are common amongst patients with spontaneous pneumothorax, especially younger patients as isolated findings, and in older patients in the context of emphysema. In patients treated according to UK clinical pathways, we now find cysts in the contralateral lung are significantly associated with increased contralateral recurrence in younger patients. We also provide a clear definition of positive CT findings assessed in this study and encourage future use of these categories.^14^^,^^15^

Our previous meta-analysis had correlated abnormalities detected by thoracic CT imaging with pneumothorax recurrence.^7^ Applying findings of that study to the UK population was challenging because studies that met our inclusion criteria showed marked differences to UK practice. For example, there was an unusually low frequency of conservative management with only 8% of cases receiving no intervention compared to 30% in recent UK series.^1^^,^^3^ This might have arisen from differences in practice between countries, or from a reporting bias caused by a relatively large number of surgical series.^6^^,^^20^ Importantly, comparing radiological changes between studies was challenging owing to variations and limitations in the imaging information made available, in particular whether pneumothoraces were ipsilateral to CT abnormalities. There were also differences in the definitions used for ‘positive’ CT findings. We therefore adhered closely to Fleischner Society guidelines when examining CT images.^15^

Despite potential biases inherent in our criteria for performing CT scanning, the non-recurrence and recurrence groups were well matched for sex, smoking status and initial management. Our current demographic findings are in keeping with our previous report of 492 unselected individuals from our pneumothorax service^1^^,^^3^ and with UK clinical guidance^4^^,^^6^ with 29% of all our patients initially managed conservatively. Thoracic surgery is not typically offered for a first pneumothorax in the UK except for patients experiencing a persistent air leak or a tension pneumothorax.^4^^,^^6^ Accordingly, primary surgery was uncommon in our cohort.

Surprisingly, in our series fewer recurrences were observed in cannabis smokers. As with our cohort, studies of cannabis smoking are frequently confounded by high rates of combined cannabis and tobacco smoking.^21^^,^^22^ Nevertheless, in keeping with our findings, cannabis smoking has been reported to be associated with more marked bullous emphysema.^22–24^ It is possible that the reduced frequency of centrilobular emphysema that we observed in cannabis smokers might reflect their lower tobacco pack-years. In contrast to our study, others have reported more frequent recurrences in cannabis smokers compared with non-smokers.^22^^,^^25^ To resolve this disparity, sufficiently powered, dedicated studies will be required to address the true association between cannabis smoking and rates of pneumothorax and recurrence. In such studies, it will also be important to obtain data on the quantities of cannabis consumed, such as joint-years, as this likely contributes to the development and severity of disease.^24^

Our patient cohort exhibited a high recurrence rate of 55%. It is our clinical practice to perform CT imaging on patients following a recurrence, those with a family history of pneumothorax, all females and patients older than 30 years of age.^1^^,^^26^ These selection criteria, together with the patient cohort being selected from a specialist tertiary referral pneumothorax centre, may account for the high frequency of recurrence. In contrast to our previous work,^1^ recurrences were similar in males and females. The exclusion of women with thoracic endometriosis in the current study may have contributed to this.

The usefulness of identifying cystic legions as a prognostic factor for pneumothorax recurrence is an open topic in the literature. Our findings agree with recent studies suggesting that cysts are a predictive factor for contralateral recurrence,^27^^,^^28^ though older studies had found no such association.^29–31^ Our study did not suggest the presence of cysts predict overall recurrence nor ipsilateral recurrence in agreement with some,^32^ but not all previous reports.^33–35^ We also observe contralateral cyst size to be associated with contralateral recurrence as seen previously,^36^ though this failed to persist in Cox regression analysis as was seen by others.^37^

Existing definitions of primary spontaneous pneumothorax (PSP) require the absence of underlying chronic lung disease, yet paradoxically a majority of PSP occurs in patients found to have blebs or bullae. By contrast, secondary spontaneous pneumothorax (SSP) requires the presence of underlying chronic lung disease. Since we examined associations with CT changes, we assessed pneumothorax recurrence in groups categorized principally by age, rather than using the more arbitrary PSP/SSP nomenclature. Our series included more younger individuals, which may account for the significance of cystic change being seen in that larger group. Cohorts including more older individuals would be needed to address this risk in that group with adequate power.

Pulmonary cystic change can arise from many aetiologies. In this study, we included patients with emphysematous features like blebs and bullae, and patients with parenchyma cystic lesions such as those seen in Birt–Hogg–Dubé syndrome. We chose this approach to ensure our data remain applicable to undifferentiated real-world clinical scenarios. There is unavoidable morphological overlap between ‘cystic’ lesions (blebs, bullae, cystic lung disease) and specific aetiologies are difficult to elicit through CT scans alone.^15^ Hence, we believe that our inclusion of ‘cystic’ lesions generally enables our data to be more applicable for clinical use.

There are several limitations to this study. Our specialist pneumothorax service may be skewed towards patients with familial disease and cystic pathology, overestimating the usefulness of CT changes in predicting recurrence. In addition, there was a preponderance of younger patients in our cohort, which may reflect the know epidemiology of pneumothorax with a relative deficit of older smokers being seen in southern and rural parts of the UK.^38^ A majority of patients did not have a thoracic CT scan performed prior to their first recurrence (68%). Analysis of these ‘post-recurrence’ CT scans was conducted assuming that lung parenchymal changes remain relatively static. This is likely to be true for patients undergoing CT scanning shortly after their first recurrence. However, a minority of cases had CT scans performed after a prolonged delay. Newly formed blebs may increase recurrence risk^39–41^ and so scans performed immediately after a first pneumothorax might be less likely to identify those at risk of contralateral recurrence if cysts develop at a later date. Currently, use of a thoracic CT scan does not feature in the management pathway for PSP^4^ and so prolonged prospective analysis would be required to address this limitation. Current clinical pathways may also contribute to introducing ascertainment bias since patients suffering recurrences are more likely to be scanned. Of 287 patients with pneumothorax who were eligible for analysis (i.e. those without trauma, infection, thoracic endometriosis, malignancy, interstitial lung disease or congenital abnormality were excluded), 43 (15%) did not have a CT scan. These were young males with no family history of pneumothorax who did not suffer a recurrence. This may explain the relatively high recurrence rate in our cohort. Our cohort is skewed to younger individuals and so is likely underpowered to report risk of recurrence in the older group. Although our regional service frequently receives notification when our patients suffer recurrences under the care of other hospitals, we did not have ethical approval to contact historical cases and so may have underestimate the recurrence rates.

In conclusion, we showed that contralateral lung cysts in younger individuals are associated with a higher risk of contralateral recurrences. This suggests that pulmonary cysts and pneumothorax are causally linked, either by cyst rupture leading to pneumothorax or from them sharing a common aetiological mechanism. Our study supports the rationale for conducting a prospective study of the utility of thoracic CT in predicting recurrence rates in pneumothorax.

## Supplementary Material

hcae129_Supplementary_Data

## References

[1] Grimes HL , Holden S, Babar J, Karia S, Wetscherek MT, Barker A, et al Combining clinical, radiological and genetic approaches to pneumothorax management. Thorax 2022; 77:196–8. 34145047 10.1136/thoraxjnl-2021-217210 PMC8762013

[2] Brown SGA , Ball EL, Perrin K, Asha SE, Braithwaite I, Egerton-Warburton D, et al Conservative versus interventional treatment for spontaneous pneumothorax. N Engl J Med 2020; 382:405–15. 31995686 10.1056/NEJMoa1910775

[3] Nikolic MZ , Lok LS, Mattishent K, Barth S, Yung B, Cummings NM, et al Noninterventional statistical comparison of BTS and CHEST guidelines for size and severity in primary pneumothorax. Eur Respir J 2015; 45:1731–4. 25792629 10.1183/09031936.00118614 PMC4450154

[4] Roberts ME , Rahman NM, Maskell NA, Bibby AC, Blyth KG, Corcoran JP, et al British thoracic society guideline for pleural disease. Thorax 2023; 78:s1–42. 10.1136/thorax-2022-219784 37433578

[5] Rintoul RC , Marciniak SJ. What's new in pleural disease? Thorax 2023; 78:1057–8. 37848216 10.1136/thorax-2022-219630

[6] MacDuff A , Arnold A, Harvey J, BTS Pleural Disease Guideline Group. Management of spontaneous pneumothorax: British Thoracic Society Pleural Disease Guideline 2010. Thorax 2010; 65 Suppl 2:ii18–31. 20696690 10.1136/thx.2010.136986

[7] Girish M , Pharoah PD, Marciniak SJ. Meta-analysis of the association between emphysematous change on thoracic computerized tomography scan and recurrent pneumothorax. QJM 2022; 115:215–21. 33538832 10.1093/qjmed/hcab020 PMC9020478

[8] West JB. Distribution of mechanical stress in the lung, a possible factor in localisation of pulmonary disease. Lancet 1971; 1:839–41. 4102531 10.1016/s0140-6736(71)91501-7

[9] Lippert HL , Lund O, Blegvad S, Larsen HV. Independent risk factors for cumulative recurrence rate after first spontaneous pneumothorax. Eur Respir J 1991; 4:324–31. 1864347

[10] Voge VM , Anthracite R. Spontaneous pneumothorax in the USAF aircrew population: a retrospective study. Aviat Space Environ Med 1986; 57:939–49. 3778392

[11] Walker SP , Bibby AC, Halford P, Stadon L, White P, Maskell NA. Recurrence rates in primary spontaneous pneumothorax: a systematic review and meta-analysis. Eur Respir J 2018; 52:1800864. 30002105 10.1183/13993003.00864-2018

[12] Hallifax RJ , Goldacre R, Landray MJ, Rahman NM, Goldacre MJ. Trends in the incidence and recurrence of inpatient-treated spontaneous pneumothorax, 1968–2016. JAMA 2018; 320:1471–80. 30304427 10.1001/jama.2018.14299 PMC6233798

[13] Tschopp JM , Bintcliffe O, Astoul P, Canalis E, Driesen P, Janssen J, et al ERS task force statement: diagnosis and treatment of primary spontaneous pneumothorax. Eur Respir J 2015; 46:321–35. 26113675 10.1183/09031936.00219214

[14] Lynch DA , Austin JH, Hogg JC, Grenier PA, Kauczor HU, Bankier AA, et al CT-definable subtypes of chronic obstructive pulmonary disease: a statement of the Fleischner society. Radiology 2015; 277:192–205. 25961632 10.1148/radiol.2015141579 PMC4613878

[15] Bankier AA , MacMahon H, Colby T, Gevenois PA, Goo JM, Leung ANC, et al Fleischner Society: glossary of terms for thoracic imaging. Radiology 2024; 310:e232558. 38411514 10.1148/radiol.232558 PMC10902601

[16] Team RC. R: A Language and Environment for Statistical Computing. R Foundation for Statistical Computing, Vienna, Austria. https://wwwR-projectorg/. 2021.

[17] Team R. RStudio: Integrated Development for R. RStudio, PBC, Boston, MA. http://wwwrstudiocom/. 2020.

[18] Wickham H , Averick M, Bryan J, Chang W, McGowan L, François R, et al Welcome to the Tidyverse. JOSS 2019; 4:1686.

[19] Balduzzi S , Rucker G, Schwarzer G. How to perform a meta-analysis with R: a practical tutorial. Evid Based Ment Health 2019; 22:153–60. 31563865 10.1136/ebmental-2019-300117 PMC10231495

[20] Baumann MH , Strange C, Heffner JE, Light R, Kirby TJ, Klein J, et al Management of spontaneous pneumothorax: an American College of Chest Physicians Delphi consensus statement. Chest 2001; 119:590–602. 11171742 10.1378/chest.119.2.590

[21] Murtha L , Sathiadoss P, Salameh JP, McInnes MDF, Revah G. Chest CT findings in marijuana smokers. Radiology 2023; 307:e212611. 36378033 10.1148/radiol.212611

[22] Stefani A , Aramini B, Baraldi C, Pellesi L, Della Casa G, Morandi U, et al Secondary spontaneous pneumothorax and bullous lung disease in cannabis and tobacco smokers: a case-control study. PLoS One 2020; 15:e0230419. 32226050 10.1371/journal.pone.0230419 PMC7105102

[23] Underner M , Urban T, Perriot J, Peiffer G, Harika-Germaneau G, Jaafari N. Spontaneous pneumothorax and lung emphysema in cannabis users. Rev Pneumol Clin 2018; 74:400–15. 30420278 10.1016/j.pneumo.2018.06.003

[24] Beshay M , Kaiser H, Niedhart D, Reymond MA, Schmid RA. Emphysema and secondary pneumothorax in young adults smoking cannabis. Eur J Cardiothorac Surg 2007; 32:834–8. 17931876 10.1016/j.ejcts.2007.07.039

[25] Hedevang Olesen W , Katballe N, Sindby JE, Titlestad IL, Andersen PE, Ekholm O, et al Cannabis increased the risk of primary spontaneous pneumothorax in tobacco smokers: a case-control study. Eur J Cardiothorac Surg 2017; 52:679–85. 28605480 10.1093/ejcts/ezx160

[26] Scott RM , Henske EP, Raby B, Boone PM, Rusk RA, Marciniak SJ. Familial pneumothorax: towards precision medicine. Thorax 2018; 73:270–6. 29288214 10.1136/thoraxjnl-2017-211169

[27] Noh D , Keum DY, Park CK. Outcomes of contralateral bullae in primary spontaneous pneumothorax. Korean J Thorac Cardiovasc Surg 2015; 48:393–7. 26665105 10.5090/kjtcs.2015.48.6.393 PMC4672973

[28] Chen YY , Huang HK, Chang H, Lee SC, Huang TW. Postoperative predictors of ipsilateral and contralateral recurrence in patients with primary spontaneous pneumothorax. J Thorac Dis 2016; 8:3217–24. 28066601 10.21037/jtd.2016.11.33 PMC5179450

[29] Mitlehner W , Friedrich M, Dissmann W. Value of computer tomography in the detection of bullae and blebs in patients with primary spontaneous pneumothorax. Respiration 1992; 59:221–7. 1485007 10.1159/000196062

[30] Smit HJ , Wienk MA, Schreurs AJ, Schramel FM, Postmus PE. Do bullae indicate a predisposition to recurrent pneumothorax? Br J Radiol 2000; 73:356–9. 10844859 10.1259/bjr.73.868.10844859

[31] Martinez-Ramos D , Angel-Yepes V, Escrig-Sos J, Miralles-Tena JM, Salvador-Sanchis JL. Usefulness of computed tomography in determining risk of recurrence after a first episode of primary spontaneous pneumothorax: therapeutic implications. Arch Bronconeumol 2007; 43:304–8. 17583639 10.1016/s1579-2129(07)60075-5

[32] Park S , Jang HJ, Song JH, Bae SY, Kim H, Nam SH, et al Do blebs or bullae on high-resolution computed tomography predict ipsilateral recurrence in young patients at the first episode of primary spontaneous pneumothorax? Korean J Thorac Cardiovasc Surg 2019; 52:91–9. 31089446 10.5090/kjtcs.2019.52.2.91 PMC6493259

[33] Primavesi F , Jager T, Meissnitzer T, Buchner S, Reich-Weinberger S, Ofner D, et al First episode of spontaneous pneumothorax: CT-based scoring to select patients for early surgery. World J Surg 2016; 40:1112–20. 26669786 10.1007/s00268-015-3371-3

[34] Casali C , Stefani A, Ligabue G, Natali P, Aramini B, Torricelli P, et al Role of blebs and bullae detected by high-resolution computed tomography and recurrent spontaneous pneumothorax. Ann Thorac Surg 2013; 95:249–55. 22785214 10.1016/j.athoracsur.2012.05.073

[35] Asano H , Ohtsuka T, Noda Y, Kato D, Mori S, Nakada T, et al Risk factors for recurrence of primary spontaneous pneumothorax after thoracoscopic surgery. J Thorac Dis 2019; 11:1940–4. 31285887 10.21037/jtd.2019.04.105 PMC6588783

[36] Yang J , Hu X, Li J, Zhang G, Ge Y, Wei W. Correlative analysis of lung CT findings in patients with Birt–Hogg–Dube syndrome and the occurrence of spontaneous pneumothorax: a preliminary study. BMC Med Imaging 2022; 22:22. 35125098 10.1186/s12880-022-00743-3 PMC8819866

[37] Olesen WH , Katballe N, Sindby JE, Titlestad IL, Andersen PE, Lindahl-Jacobsen R, et al Surgical treatment versus conventional chest tube drainage in primary spontaneous pneumothorax: a randomized controlled trial. Eur J Cardiothorac Surg 2018; 54:113–21. 29509892 10.1093/ejcts/ezy003

[38] Gupta D , Hansell A, Nichols T, Duong T, Ayres JG, Strachan D. Epidemiology of pneumothorax in England. Thorax 2000; 55:666–71. 10899243 10.1136/thorax.55.8.666 PMC1745823

[39] Choi SY , Kim DY, Suh JH, Yoon JS, Jeong JY, Park CB. New bullae formation in the staple line increases the risk of recurrent pneumothorax following video-assisted thoracoscopic surgery bullectomy for primary spontaneous pneumothorax. J Thorac Dis 2018; 10:4287–92. 30174875 10.21037/jtd.2018.06.07 PMC6105948

[40] Onuki T , Kawamura T, Kawabata S, Yamaoka M, Inagaki M. Neo-generation of neogenetic bullae after surgery for spontaneous pneumothorax in young adults: a prospective study. J Cardiothorac Surg 2019; 14:20. 30674336 10.1186/s13019-019-0848-4 PMC6344986

[41] Tsuboshima K , Matoba Y, Wakahara T. Contralateral bulla neogenesis associated with postoperative recurrences of primary spontaneous pneumothorax in young patients. J Thorac Dis 2019; 11:5124–9. 32030229 10.21037/jtd.2019.12.05 PMC6988004

