# Supplementary material for: CT features associated with contralateral recurrence of spontaneous pneumothorax
Source: QJM. 2024 Jul 8;117(12):837–45. doi: 10.1093/qjmed/hcae129 (PMC11760504; doi:10.1093/qjmed/hcae129)
Supplement: hcae129_Supplementary_Data [file hcae129_supplementary_data.zip › hcae129_Supplementary_Data/Acronyms_2023_SM1.docx]

**Acronyms**

British Thoracic Society BTS

Computerised Tomography CT

Confidence Interval CI

Hazard Ratio HR

Primary Spontaneous Pneumothorax PSP

Secondary Spontaneous Pneumothorax SSP
