# Supplementary material for: CT features associated with contralateral recurrence of spontaneous pneumothorax
Source: QJM. 2024 Jul 8;117(12):837–45. doi: 10.1093/qjmed/hcae129 (PMC11760504; doi:10.1093/qjmed/hcae129)
Supplement: hcae129_Supplementary_Data [file hcae129_supplementary_data.zip › hcae129_Supplementary_Data/CT_PSP_Supplementary material_SM2.pdf]

CT features associated with contralateral recurrence of spontaneous pneumothorax.

Lance A Burn**,** Maria TA Wetscherek, Paul D Pharoah, Stefan J. Marciniak

Supplementary information

Table S1: Demographics of Included (having CT) vs Excluded (lacking CT).

	Included (n = 243)	Excluded, no-CT (n = 43)	Chi^2 test (P-value)
Male sex – N (%)	189 (78%)	40 (93%)	**0.036**
Age – median (range)	30.2 (13.4-95.1)	21.4 (15.2-76.4)	**<0.001**[%]
Deceased – N (%)	6 (2.5%)	0 (0%)	0.64
**Smoking status** – N (%)			
Ever	142 (58%)	21 (49%)	0.32
Current	70 (29%)	16 (37%)	**0.038**
**Smoking type** – N (%)			
Tobacco	140 (58%)	20 (47%)	0.24
Cannabis	76 (31%)	11 (26%)	0.56
Crack cocaine	1 (0.4%)	0 (0%)	1.00
Hooka	1 (0.4%)	0 (0%)	1.00
Smoking pack-years – mean (range)	9.4 (0-96)	3.3 (0-60)	**0.006**[%]
Missing data – N (%)	18 (7.4%)	6 (14%)	-
**Initial treatment** – N (%)			
Conservative	70 (29%)	25 (58%)	**<0.001**
Aspirated	35 (14%)	3 (7.0%)	0.28
Thoracic drain	106 (44%)	14 (33%)	0.24
Surgery	27 (11%)	1 (2.3%)	0.13
Missing data	5 (2.1%)	0 (0%)	-
Family history – N (%)	36 (15%)	1 (2.3%)	**0.045**
First pneumothorax – left/right	129 (53%) / 114 (47%)	28 (65%) / 15 (35%)	0.20
**Recurrence** – N (%)			
Left / right	67 (28%) / 66 (27%)	1 (2.3%) / 1 (2.3%)	1.00
Ipsilateral / contralateral	99 (41%) / 34 (14%)	0 (0%) / 2 (4.7%)	**<0.001**
**Pulmonary diagnosis** – N (%)			
Birt-Hogg-Dubé syndrome	5 (2.1%)	0 (0%)	0.75
Marfan syndrome	2 (0.8%)	0 (0%)	1.00
A1AT deficiency	2 (0.8%)	0 (0%)	1.00
Vascular Ehlers-Danlos syndrome	1 (0.4%)	0 (0%)	1.00
Other	8 (3.3%)	0 (0%)	-

[%]Mann-Whitney test

Table S2: Univariate cox regression analysis of overall recurrence), censored at 120 months

Characteristic	N	Event N	HR[1]	95% CI[1]	p-value
**Age**	243	127	0.99	0.98, 1.00	0.058
**Smoking pack-years**	225	121	0.99	0.97, 1.00	**0.040**
**Cannabis use**	242	127			
0			—	—	
1			0.66	0.44, 0.98	**0.040**
**Initial pneumothorax management**	238	123			
aspiration			—	—	
conservative			1.03	0.60, 1.79	0.90
ICD			0.89	0.53, 1.50	0.66
surgery			0.49	0.23, 1.05	0.066
**Family Hx of pneumothorax**	243	127			
0			—	—	
1			1.77	1.14, 2.73	**0.010**
**Paraseptal emphysema prescence**	243	127			
0			—	—	
1			0.89	0.63, 1.27	0.53
**Centrilobular emphysema presence**	243	127			
0			—	—	
1			0.70	0.47, 1.03	0.070
**Cyst presence**	243	127			
0			—	—	
1			1.13	0.70, 1.85	0.61
**Largest cyst across both lungs**	243	127	1.00	0.99, 1.01	0.86

[1] HR = Hazard Ratio, CI = Confidence Interval

Multivariate analysis did <u>not</u> reveal a model whereby >1 characteristic showed a P-value <0.05

Table S3: Univariate cox regression analysis of overall recurrence for ≤50-years-old cohort, censored at 120 months

Characteristic	N	Event N	HR[1]	95% CI[1]	p-value
**Age**	187	101	0.96	0.93, 0.98	**<0.001**
**Smoking pack-years**	174	96	0.95	0.91, 0.99	**0.014**
**Cannabis use**	186	101			
0			—	—	
1			0.63	0.41, 0.96	**0.031**
**Initial pneumothorax management**	185	99			
aspiration			—	—	
conservative			1.09	0.62, 1.92	0.75
ICD			0.71	0.41, 1.23	0.22
surgery			0.51	0.23, 1.18	0.12
**Family Hx of pneumothorax**	187	101			
0			—	—	
1			1.70	1.07, 2.70	**0.024**
**Paraseptal emphysema prescence**	187	101			
0			—	—	
1			0.82	0.55, 1.23	0.34
**Centrilobular emphysema presence**	187	101			
0			—	—	
1			0.51	0.29, 0.89	**0.018**
**Cyst presence**	187	101			
0			—	—	
1			1.31	0.73, 2.34	0.36
**Largest cyst across both lungs**	187	101	1.00	0.99, 1.01	0.89

[1] HR = Hazard Ratio, CI = Confidence Interval

Multivariate analysis did <u>not</u> reveal a model whereby >1 characteristic showed a P-value <0.05

Table S4: Cox regression analysis of ipsilateral recurrence, censored at 120 months

	Univariate analysis					Multivariate analysis			
**Characteristic**	**N**	**Event N**	**HR**[1]	**95% CI**[1]	**p-value**	**Event N**	**HR**[1]	**95% CI**[1]	**p-value**
**Age**	243	95	1.00	0.99, 1.01	0.94				
**Smoking pack-years**	225	91	0.99	0.98, 1.01	0.31				
**Cannabis use**	242	95				92			
0			—	—			—	—	
1			0.56	0.35, 0.91	**0.020**		0.60	0.37, 0.98	**0.042**
**Initial pneumothorax management**	238	92				92			
aspiration			—	—			—	—	
conservative			0.91	0.49, 1.71	0.77		0.91	0.48, 1.71	0.8
ICD			0.94	0.52, 1.68	0.83		0.99	0.55, 1.78	>0.9
surgery			0.25	0.08, 0.76	**0.014**		0.29	0.09, 0.86	**0.026**
**Family Hx of pneumothorax**	243	95							
0			—	—					
1			1.44	0.85, 2.45	0.17				
**Ipsilateral paraseptal emphysema**	243	95							
0			—	—					
1			0.89	0.59, 1.34	0.56				
**Ipsilateral centrilobular emphysema**	243	95							
0			—	—					
1			0.70	0.39, 1.27	0.24				
2			0.76	0.35, 1.65	0.48				
3			1.44	0.69, 3.00	0.33				
**Ipsilateral cyst number**	243	95							
0			—	—					
1			0.64	0.38, 1.06	0.082				
2			0.70	0.42, 1.16	0.16				
**Ipsilateral largest cyst size**	243	95	1.00	0.99, 1.01	0.71				

[1] HR = Hazard Ratio, CI = Confidence Interval

Table S5: Univariate cox regression analysis of ipsilateral recurrence for ≤50-years-old cohort, censored at 120 months.

Characteristic	N	Event N	HR[1]	95% CI[1]	p-value
**Age**	187	71	0.97	0.95, 1.00	0.068
**Smoking pack-years**	174	68	0.95	0.90, 1.00	**0.046**
**Cannabis use**	186	71			
0			—	—	
1			0.57	0.34, 0.95	**0.032**
**Initial pneumothorax management**	185	70			
aspiration			—	—	
conservative			0.90	0.47, 1.73	0.76
ICD			0.70	0.38, 1.31	0.27
surgery			0.25	0.07, 0.86	**0.028**
**Family Hx of pneumothorax**	187	71			
0			—	—	
1			1.54	0.88, 2.71	0.13
**Ipsilateral paraseptal emphysema**	187	71			
0			—	—	
1			0.72	0.44, 1.20	0.21
**Ipsilateral centrilobular emphysema**	187	71			
0			—	—	
1			0.53	0.24, 1.15	0.11
2			0.00	0.00, Inf	>0.99
3					
**Ipsilateral cyst number**	187	71			
0			—	—	
1			0.69	0.39, 1.21	0.19
2			0.63	0.34, 1.20	0.16
**Ipsilateral largest cyst size**	187	71	1.00	0.98, 1.01	0.66

[1] HR = Hazard Ratio, CI = Confidence Interval

Multivariate analysis did not reveal a model whereby >1 characteristic showed a P-value <0.05

Table S6: Cox regression analysis of contralateral recurrence, censored at 120 months

	Univariate analysis					Multivariate analysis			
**Characteristic**	**N**	**Event N**	**HR**[1]	**95% CI**[1]	**p-value**	**Event N**	**HR**[1]	**95% CI**[1]	**p-value**
**Age**	243	32	0.94	0.90, 0.97	**<0.001**	32	0.94	0.90, 0.97	**0.001**
**Smoking pack-years**	225	30	0.93	0.87, 0.99	**0.026**				
**Cannabis use**	242	32							
0			—	—					
1			0.98	0.47, 2.03	0.95				
**Initial pneumothorax management**	238	31							
aspiration			—	—					
conservative			1.51	0.49, 4.69	0.48				
ICD			0.70	0.22, 2.27	0.55				
surgery			1.33	0.37, 4.71	0.66				
**Family Hx of pneumothorax**	243	32							
0			—	—					
1			2.98	1.37, 6.51	**0.006**				
**Contralateral paraseptal emphysema**	243	32							
0			—	—					
1			0.91	0.45, 1.85	0.80				
**Contralateral centrilobular emphysema**	243	32							
0			—	—					
1			0.00	0.00, Inf	>0.99				
2			1.19	0.46, 3.09	0.72				
3			0.00	0.00, Inf	>0.99				
**Contralateral cyst number**	243	32				32			
0			—	—			—	—	
1			8.31	1.94, 35.6	**0.004**		7.07	1.65, 30.3	**0.008**
2			3.53	0.77, 16.1	0.10		5.44	1.17, 25.2	**0.030**
**Contralateral largest cyst size**	243	32	1.00	0.99, 1.02	0.85				

[1] HR = Hazard Ratio, CI = Confidence Interval

Table S7: Cox regression analysis of contralateral recurrence for ≤50-years-old cohort, censored at 120 months.

	Univariate analysis					Multivariate analysis			
**Characteristic**	**N**	**Event N**	**HR[1]**	**95% CI[1]**	**p-value**	**Event N**	**HR[1]**	**95% CI[1]**	**p-value**
**Age**	187	30	0.91	0.86, 0.96	**<0.001**	30	0.88	0.82, 0.94	**<0.001**
**Smoking pack-years**	174	28	0.94	0.86, 1.02	0.14				
**Cannabis use**	186	30							
0			—	—					
1			0.78	0.37, 1.65	0.52				
**Initial pneumothorax management**	185	29							
aspiration			—	—					
conservative			1.82	0.59, 5.66	0.30				
ICD			0.72	0.22, 2.41	0.60				
surgery			1.48	0.40, 5.52	0.56				
**Family Hx of pneumothorax**	187	30							
0			—	—					
1			2.13	0.94, 4.83	0.069				
**Contralateral paraseptal emphysema**	187	30							
0			—	—					
1			1.09	0.52, 2.27	0.82				
**Contralateral centrilobular emphysema**	187	30							
0			—	—					
1			0.00	0.00, Inf	>0.99				
2			2.55	0.89, 7.33	0.081				
3									
**Contralateral cyst number**	187	30				30			
0			—	—			—	—	
1			7.01	1.64, 30.0	**0.009**		5.13	1.17, 22.6	**0.030**
2			3.65	0.78, 17.2	0.10		1.96	0.32, 11.9	0.5
**Contralateral largest cyst size**	187	30	1.02	1.0, 1.04	0.14	30	1.05	1.01, 1.09	**0.006**

[1] HR = Hazard Ratio, CI = Confidence Interval

Table S8: Demographics table of smokers, categorised by cannabis use

	Tobacco-only users (n = 65)	Cannabis users (n = 76)	Chi^2 test (P-value)
Male sex – N (%)	51 (78%)	65 (86%)	0.38
Age – median (range)	63.1 (14.9-89.4)	29.4 (17-68.2)	**<0.001**[%]
Deceased* – N (%)	5 (7.7%)	0 (0%)	**0.045**
Smoking status – N (%)			
Ever	65 (100%)	76 (100%)	1.00
Current	19 (29%)	50 (66%)	**<0.001**
Smoking type – N (%)			
Tobacco	65 (100%)	74 (97%)	0.55
Smoking pack-years – mean (range)	25.6 (0.05-96)	8.89 (0-70)	**<0.001**[%]
Missing data – N (%)	9 (14%)	8 (11%)	-
Initial treatment – N (%)			
Conservative	17 (26%)	18 (24%)	0.89
Aspirated	5 (7.7%)	8 (11%)	0.77
Thoracic drain	35 (54%)	38 (50%)	0.77
Surgery	6 (9.2%)	11 (14%)	0.49
Missing data	2 (3.1%)	1 (1.3%)	-
Family history[#] – N (%)	7 (11%)	5 (6.6%)	0.56
First pneumothorax – left/right	28 (43%) / 37 (57%)	32 (42%) / 44 (58%)	1.00
Recurrence – N (%)			
Left / right	12 (18%) / 26 (40%)	17 (22%) / 16 (21%)	0.14
Ipsilateral / contralateral	33 (51%) / 5 (7.7%)	22 (29%) / 11 (14%)	0.081
Pulmonary diagnosis – N (%)			
Birt-Hogg-Dubé syndrome	1 (1.5%)	0 (0%)	0.94
Marfan syndrome	1 (1.5%)	0 (0%)	0.94
A1AT deficiency	2 (3.1%)	0 (0%)	0.41
Vascular Ehlers-Danlos syndrome	0 (0%)	0 (0%)	1.00
Other	1 (1.5%)	3 (3.9%)	-

[%]Mann-Whitney test, n = 1 patient missing from table due to unknown smoking / cannabis use status

Table S9: CT changes in tobacco versus cannabis users

Parenchymal change	Tobacco-only users (n = 65)	Cannabis users (n = 76)	Chi^2 test (P-value)
Ipsilateral – N (%)			
Isolated blebs / bullae	7 (11%)	14 (17%)	0.30
Emphysema	51 (78%)	57 (75%)	0.78
Centrilobular	47 (72%)	25 (33%)	**<0.001**
Paraseptal	36 (55%)	57 (75%)	**0.014**
Cystic lung disease	1 (1.5%)^	0 (0%)	0.94
No abnormality	6 (9.2%)	5 (6.6%)	0.79
Contralateral – N (%)			
Isolated blebs / bullae	5 (7.7%)	8 (11%)	0.77
Emphysema	54 (83%)	58 (76%)	0.43
Centrilobular	50 (77%)	27 (36%)	**<0.001**
Paraseptal	35 (54%)	56 (74%)	**0.023**
Cystic lung disease	1 (1.5%)^	0 (0%)	0.94
No abnormality	5 (7.7%)	10 (13%)	0.44

^n=1 patient also had centrilobular and paraseptal emphysema
